# A Missense Mutation in the Transcription Factor ETV5 Leads to Sterility, Increased Embryonic and Perinatal Death, Postnatal Growth Restriction, Renal Asymmetry and Polydactyly in the Mouse

**DOI:** 10.1371/journal.pone.0077311

**Published:** 2013-10-21

**Authors:** Duangporn Jamsai, Brett J. Clark, Stephanie J. Smith, Belinda Whittle, Christopher C. Goodnow, Christopher J. Ormandy, Moira K. O’Bryan

**Affiliations:** 1 The Department of Anatomy and Developmental Biology, Monash University, Melbourne, Victoria, Australia; 2 Australian Phenomics Facility, The Australian National University, Canberra, Australian Capital Territory, Australia; 3 The Garvan Institute of Medical Research, Sydney, New South Wales, Australia; McGill University, Canada

## Abstract

ETV5 (Ets variant gene 5) is a transcription factor that is required for fertility. In this study, we demonstrate that ETV5 plays additional roles in embryonic and postnatal developmental processes in the mouse. Through a genome-wide mouse mutagenesis approach, we generated a sterile mouse line that carried a nonsense mutation in exon 12 of the *Etv5* gene. The mutation led to the conversion of lysine at position 412 into a premature termination codon (PTC) within the ETS DNA binding domain of the protein. We showed that the PTC-containing allele produced a highly unstable mRNA, which in turn resulted in an undetectable level of ETV5 protein. The *Etv5* mutation resulted in male and female sterility as determined by breeding experiments. Mutant males were sterile due to a progressive loss of spermatogonia, which ultimately resulted in a Sertoli cell only phenotype by 8 week-of-age. Further, the ETV5 target genes *Cxcr4* and *Ccl9* were significantly down-regulated in mutant neonate testes. CXCR4 and CCL9 have been implicated in the maintenance and migration of spermatogonia, respectively. Moreover, the *Etv5* mutation resulted in several developmental abnormalities including an increased incidence of embryonic and perinatal lethality, postnatal growth restriction, polydactyly and renal asymmetry. Thus, our data define a physiological role for ETV5 in many aspects of development including embryonic and perinatal survival, postnatal growth, limb patterning, kidney development and fertility.

## Introduction

The transcription of genes is controlled by proteins known as transcription factors. These factors have fundamental roles in all developmental processes, and mutations that affect transcription factor function have been shown to be associated with many human diseases [1].

The ETS (E-twenty six) family is one of the largest families of transcription factors. They play critical roles in various aspects of cell physiology including proliferation, differentiation, migration, cell-cell interaction, apoptosis and oncogenesis [2], [3]. All ETS members share an evolutionarily conserved DNA binding domain of ∼85 amino acids known as the ETS domain, which binds to a consensus purine-rich motif sequence (5′-GGA(A/T)-3′) within the promoters of target genes [2]. The majority of ETS proteins acts as transcriptional activators while a few members act as transcriptional repressors [2], [3]. ETS proteins activate or repress transcription of target genes in cooperation with other transcription factors and/or co-factors in order to enhance the specificity of promoter binding sites [2].

The ETS family members are subdivided into 12 subfamilies based on their sequence similarities [2]. ETV5 is a member of the PEA3 subfamily, which is composed of three members: ETV1 (alias ER81); ETV4 (alias PEA3 and E1AF); and ETV5. ETV5 has a widespread expression profile in developing and adult tissues [4]–[6], including the testis [7], [8] and ovary [9]. In mouse and human testes, ETV5 is localized to Sertoli cells and germ cells including spermatogonia [8], [10]. Mouse model studies indicate that ETV5 is essential for male [7], [10], [11] and female [12] fertility. Homozygous deletion of exons 2–6 of the mouse *Etv5* gene (the allele referred to as *Etv5^tm1Kmm^*) resulted in the progressive loss of male germ cells following the first wave of spermatogenesis and ultimately led to a complete loss of all germ cells and sterility in adulthood [7], [10], [11]. In humans, this phenotype is referred to as “Sertoli cell only” (SCO) syndrome [13]. ETV5 has been shown to play a critical role in the establishment of the spermatogonia stem cell (SSC) pool and the homeostasis of SSC self-renewal and differentiation [7], [14], [15]. Consistent with this observation, putative splicing mutations in the human *ETV5* gene have been associated with human SCO [8]. In the ovary, ETV5 is localized to granulosa and cumulus cells [9]. *Etv5^tm1Kmm^* homozygous females are sterile due to defects in oocyte development, decreased ovulation and mating rates [12].

In addition, ETV4 and ETV5 have been shown to have a redundant role in kidney branching morphogenesis via the GDNF-RET pathway [16]. Severely compromised ETV4 and ETV5 expression resulted in the complete failure of kidney development in the mouse [16]. Similarly, ETV4 and ETV5 have been shown to play a critical role in the outgrowth of anterior-posterior limbs in the mouse via the Sonic Hedgehog (Shh) pathways [17], [18].

In this study, we report the generation of an *Etv5* mutant mouse line via a large-scale N-ethyl-N-nitrosourea (ENU) mutagenesis screen for sterility-causing genes. Our data further confirm an essential role for ETV5 in fertility. We demonstrate that the ENU-induced *Etv5* mutation resulted in the production of a severe loss-of-function allele, which leads to developmental abnormalities including an increase incidence of embryonic and perinatal death, postnatal growth restriction, polydactyly and renal asymmetry.

## Results and Discussion

### Etv5 Mutant Mice are Sterile

**Figure 1:**
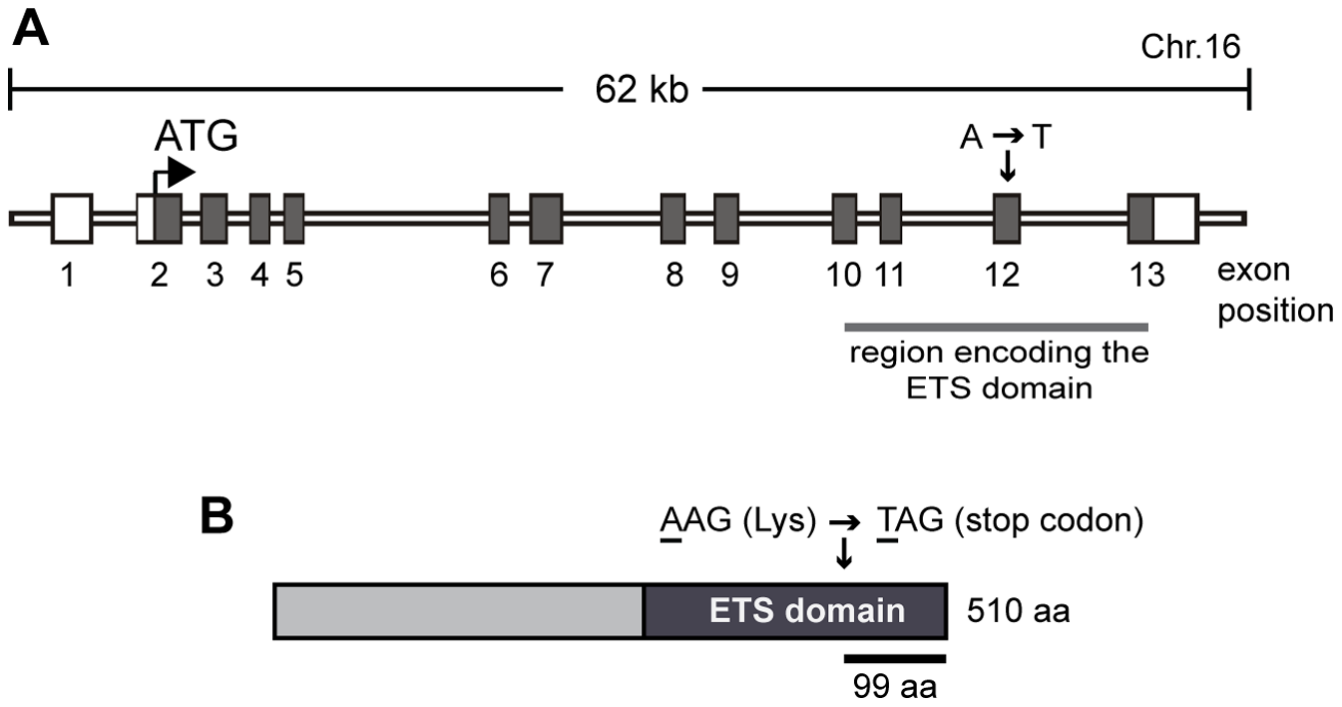
The SCO mouse line carries a missense mutation within the *Etv5* gene. (A) Schematic of the mouse *Etv5* gene and the location of the identified mutation. Exon positions are based on the ENSMUST00000079601 transcript. (B) ETV5 protein and the location of the missense amino acid.

To discover genes and pathways that are essential for male fertility, we conducted a genome-wide ENU mutagenesis screen in the mouse as previously described [19]–[21]. Using a three-generation breeding strategy to enrich for the identification of recessive mutations, we generated several sterile mouse lines including the “SCO” line. The chromosomal region containing the mutated gene in the SCO line was mapped using single nucleotide polymorphism (SNP)-based methods and ultimately narrowed to a linkage interval on chromosome 16 between SNP markers rs4165081 and rs4165422, which contained 77 genes (Ensembl release 60). Of these, one gene, *Etv5*, had previously been demonstrated to play a crucial role male fertility in mice [7]. Thus, *Etv5* was subjected to sequencing. We identified an A→T mutation within exon 12 of the *Etv5* gene (Fig. 1A), which resulted in the conversion of lysine (K) at position 412 into a premature termination codon (PTC) i.e. AAG→TAG within the EST domain of the ETV5 protein (Fig. 1B). The PTC is predicted to result in the truncation of 99 amino acids C-terminal of the ETV5 protein (Fig. 1B).

Homozygous *Etv5* mutant males (hereafter referred to as *Etv5^sco/sco^,* where sco refers to the Sertoli cell only phenotype described herein) were sterile as determined by breeding experiments. Eight-weeks-old *Etv5^sco/sco^* males and wild-type (WT) littermates (*n* = 6 per group) were mated with WT females over a period of 3 months. No pups were obtained from *Etv5^sco/sco^* male x WT female breeding pairs compared to that of 6–10 pups per litter from WT x WT breeding pairs.

**Figure 2:**
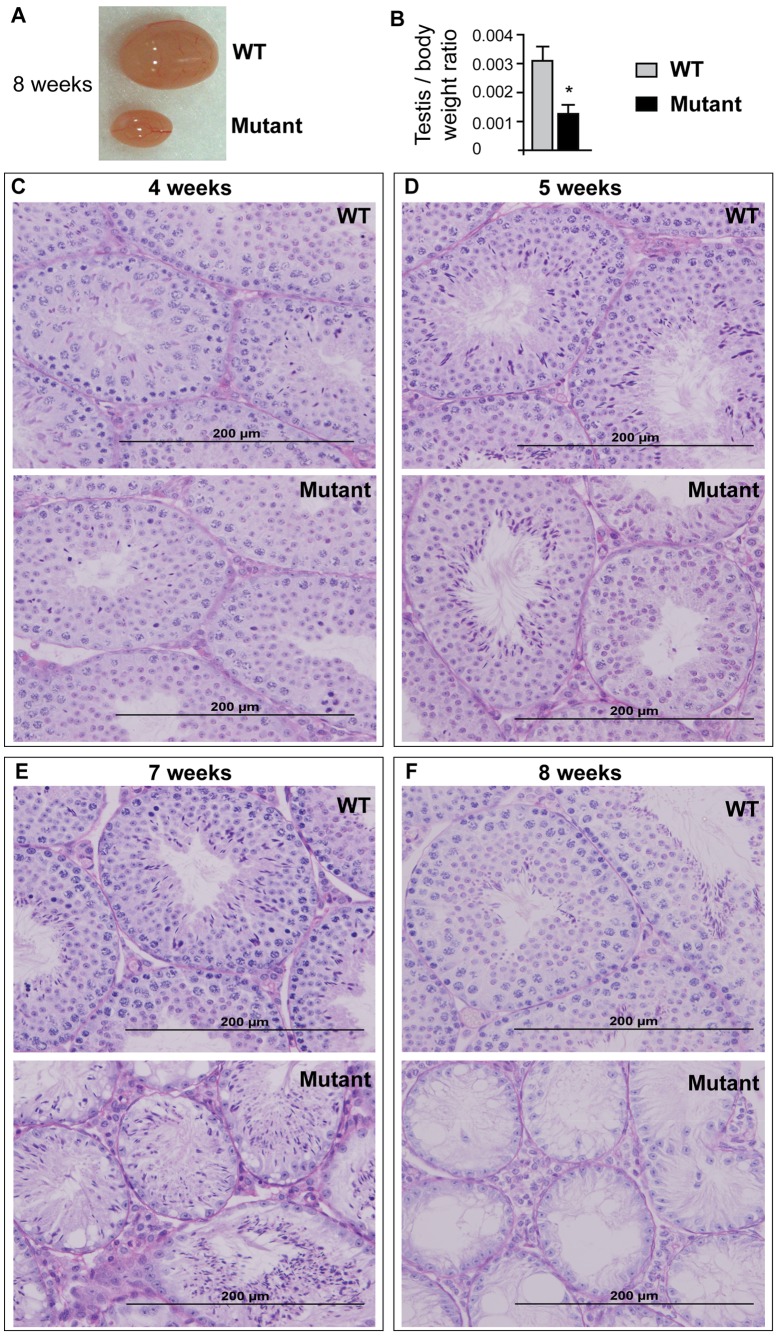
*Etv5* mutant (*Etv5^sco/sco^*) males are sterile due to the progressive loss of germ cells. (A) Testis of 8 weeks-old *Etv5^sco/sco^* and a wild-type (WT) littermate. (B) Testis weight to body weight ratio of *Etv5^sco/sco^* mice compared to WT littermates. *n* = 12 per group. *p<0.05 (unpaired *t*-test, two-tailed). (C–F) PAS staining of 4–8 weeks-old testes of *Etv5^sco/sco^* and WT mice.

To define the cause of male sterility, *Etv5^sco/sco^* males and WT littermates were culled at 4–8 weeks postnatal for testis histological analysis. At 8 week-of-age, *Etv5^sco/sco^* mice have a dramatic reduction in testis size compared to WT littermates (Fig. 2A). Consistently, the testis to body weight ratio of *Etv5^sco/sco^* males was significantly reduced compared to WT controls (p<0.001) (Fig. 2B). We subsequently examined testis histology of *Etv5^sco/sco^* and WT mice at 4, 5, 7 and 8 week-of-age. The first wave of spermatogenesis of the *Etv5^sco/sco^* mice initiated in an apparently normal way as indicated by the presence of both pre-meiotic and post-meiotic haploid germ cells at 4 week-of-age (Fig. 2C). By 5 weeks, however, the loss of germ cells was clearly discernable (Fig. 2D). Germ cell loss was pronounced at 7 weeks (Fig. 2E), and by 8 weeks a complete loss of germ cells from the seminiferous epithelium was observed (Fig. 2F). Notably, and as indicated in 7 weeks-old testis (Fig. 2E), germ cells appeared to be preferentially lost in a basal to apical direction from the seminiferous epithelium. The ultimate phenotype resembled Sertoli cell only (SCO) syndrome in humans. Thus, our data suggest that male sterility in *Etv5^sco/sco^* mice was due to the progressive loss of germ cells following the first wave of spermatogenesis. In addition to male sterility, *Etv5^sco/sco^* females were sterile. Test breeding of 6–12 weeks-old *Etv5^sco/sco^* females (*n* = 3) with WT males did not result in any pups.

The sterility phenotype observed in *Etv5^sco/sco^* mice is consistent with data previously reported from *Etv5^tm1Kmm^* homozygous males and females [7], [10]–[12], suggesting that the identified missense mutation is indeed casual of the sterility phenotype observed in the SCO mouse line. Our results however, indicated that total loss of germ cells in *Etv5^sco/sco^* males was observed earlier than that of the *Etv5^tm1Kmm^* mice i.e. by 8 weeks in *Etv5^sco/sco^* compared to 10 weeks in *Etv5^tm1Kmm^* homozygous males. These results suggest that the *Etv5^sco/sco^* allele resulted in a more severe loss-of-function allele than the *Etv5^tm1Kmm^* allele.

### The Etv5^sco/sco^ Allele Produces in a Highly Unstable Etv5 mRNA

**Figure 3:**
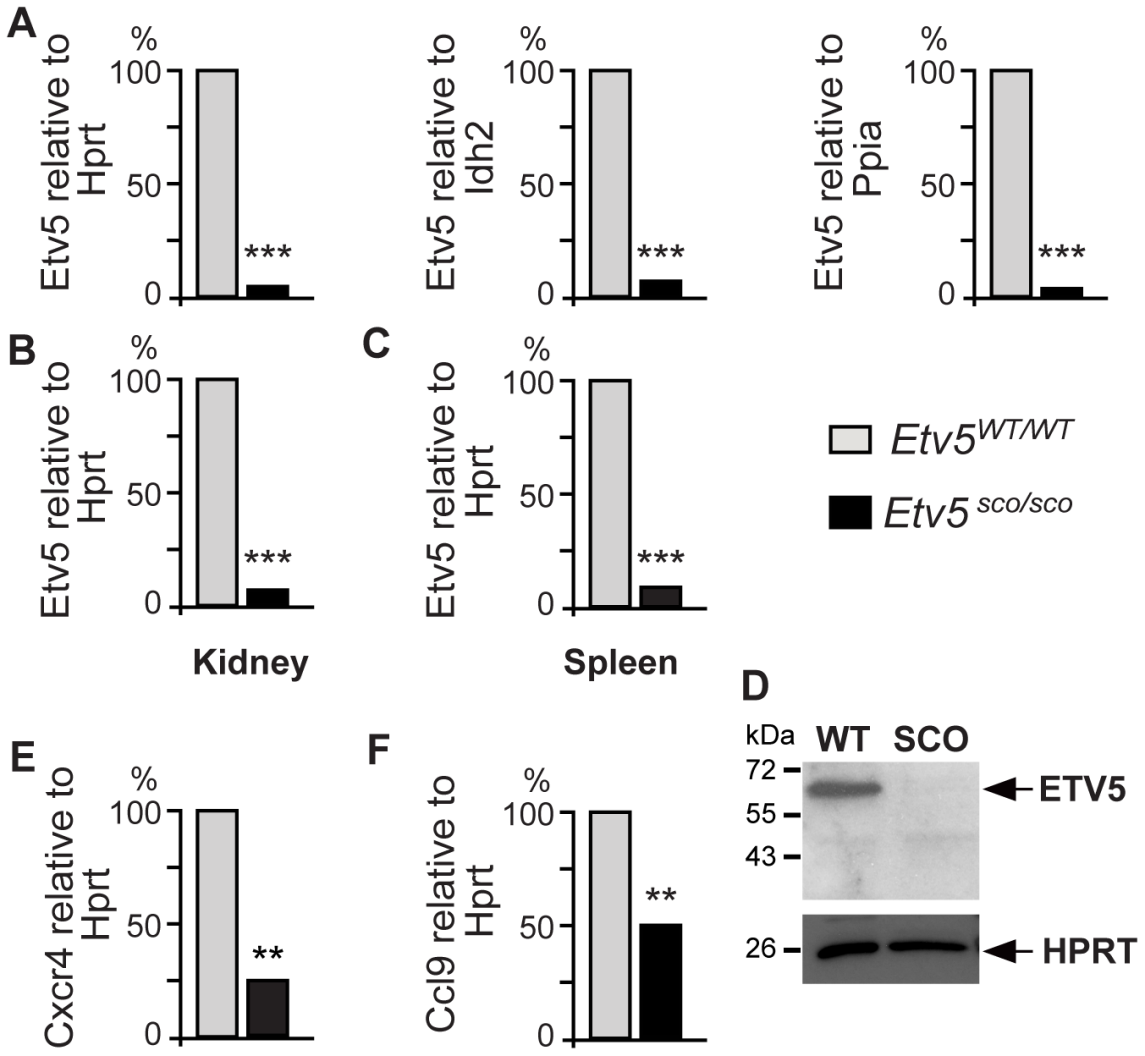
Levels of *Etv5* mRNA in postnatal day 3 testes (A), kidney (B) and spleen (C) in *Etv5^sco/sco^* and wild-type (WT) mice. (D) ETV5 immunoblotting showed an absence of full length and truncated proteins in the mutant spleen. HPRT was used as a loading control. Levels of *Cxcr4* (E) and *Ccl9* (F) mRNAs in *Etv5^sco/sco^* and WT postnatal day 3 testes. A–C, E–F, WT values were set as 100%. *n* = 3 mice per group, **p<0.01, ***p<0.001 (unpaired *t*-test, two-tailed).

As mRNAs that contain premature termination codons (PTCs) have frequently been shown to have decreased stability and were rapidly degraded through the nonsense mediated decay (NMD) pathway [22], we asked if the *Etv5^sco/sco^* allele would effect *Etv5* mRNA stability. We measured levels of *Etv5* mRNA in *Etv5^sco/sco^* and WT postnatal day 3 testes, which contains an enriched population of undifferentiated spermatogonia and comparable histology between genotypes. We used a quantitative PCR (q-PCR) assay that detected a region upstream of the mutation i.e. exons 5 and 6 of the mouse *Etv5* transcript (ENSMUST00000079601). Levels of *Etv5* mRNA were normalised against three reference mRNAs including *Hprt* (hypoxanthine guanine phosphoribosyl transferase), *Idh2* (isocitrate dehydrogenase 2 (NADP+), mitochondrial) and *Ppia* (peptidylprolyl isomerase A). Our results indicated that *Etv5^sco/sco^* testes contained a >95% reduction of *Etv5* mRNAs compared to WT males (Fig. 3A). Similarly, a significant reduction of *Etv5* mRNA was observed in other tissues including the kidney (Fig. 3B) and spleen (Fig. 3C) of *Etv5^sco/sco^* mice. These results suggest that the PTC-containing *Etv5* transcript is highly unstable.

Next, we determined, by immunoblotting, if the remaining PTC-containing *Etv5* mRNA was translated into a truncated ETV5 protein. No truncated ETV5 protein was detected in *Etv5^sco/sco^* mice (Fig. 3D), suggesting that the truncated protein was also unstable and likely to be rapidly degraded. This in turn resulted in the total absence of ETV5 protein.

### Lack of ETV5 Protein Reduces Expression of its Target Genes Cxcr4 and Ccl9 in Neonate Testes

Studies using siRNA knockdown in mouse spermatogonia cultures revealed that ETV5 regulates several genes including the CXC chemokine receptor type 4 (*Cxcr4*) [23]. CXCR4 and its chemokine ligand CXCL12 are important components within the glial cell line-derived neurotrophic factor (GDNF) pathway and have been shown to play a critical role in SSC maintenance [24], [25]. *In vivo* inhibition of CXCL12-CXCR4 signalling in adult mouse testes resulted in impaired SSC maintenance, which led to loss of the germline [25]. Further, microarray analysis of Sertoli cells obtained from the *Etv5^tm1Kmm^* homozygous mice revealed a decrease in several chemokine-encoding genes including C-c-motif ligand 9 *(Ccl9)* [26]. CCL9 and its receptor C-C-receptor type 1 (CCR1) have been implicated in SSC migration and/or retention in their microenvironment [26]. CCL9 is expressed in Sertoli cells and its expression is regulated by ETV5. CCR1 is highly expressed in gonocytes and undifferentiated spermatogonia. Impaired CCR1–CCL9 signalling via RNA interference resulted in reduced migration of spermatogonia as determined by chemotaxis assays [26]. Thus, we next investigated if the lack of ETV5 in *Etv5^sco/sco^* testis had an effect on levels of expression of *Cxcr4* and *Ccl9* during the early phase of spermatogenesis. Using qPCR analysis, we showed that levels of *Cxcr4* (Fig. 3E) and *Ccl9* (Fig. 3F) mRNAs were significantly decreased in *Etv5^sco/sco^* postnatal day 3 testes compared to WT controls. These results suggest that a progressive loss of germ cells in *Etv5^sco/sco^* mice is associated with defects in genes involved in the maintenance and migration of SSCs.

### Etv5^sco/sco^ Allele Leads to an Increased Incidence of Embryonic and Perinatal Lethality

Our breeding records also indicated that the frequency of *Etv5^sco/sco^* pups obtained from heterozygous intercrosses at weaning age (3 weeks) was significantly lower than that of the expected Mendelian ratios i.e. only ∼8% of the expected number survived to 3 weeks (p<0.001) (Table 1).

**Table 1:** Genotype frequency of pups derived from heterozygous intercrosses.

Age examined	Total number examined	*Etv5^WT/WT^*		*Etv5^WT/sco^*		*Etv5^sco/sco^*	
		% Expected	% Observed	% Expected	% Observed	% Expected	% Observed
E10.5–E13.5	95	25	24.2 (23/95)	50	52.6 (50/95)	25	23.2 (22/95)
E14.5–E18.5	85	25	27.1 (23/85)	50	57.6 (49/85)	25	15.3* (13/85)
1 week	112	25	30.3 (34/112)	50	59.0 (66/112)	25	10.7** (12/112)
3 weeks	312	25	33.0 (103/312)	50	58.7 (183/312)	25	8.3%** (26/312)

* p<0.05;

** p<0.001 (unpaired t-test, two-tailed). A shift in the ratio of *Etv5^WT/WT^* and *Etv5^WT/sco^* mice obtained at E14.5–E18.5, 1 week and 3 weeks was due to the lost of *Etv5^sco/sco^* mice.

**Figure 4:**
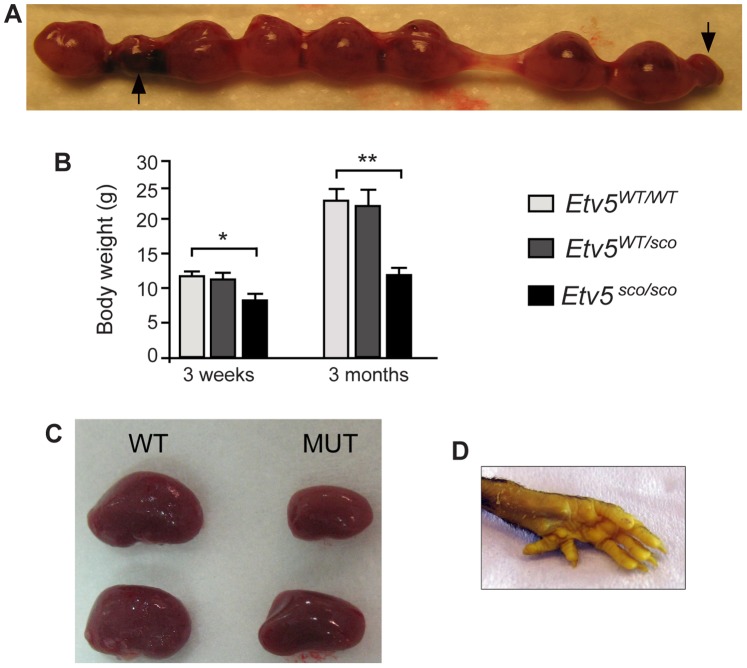
*Etv5^sco/sco^* mice exhibit several developmental abnormalities. (A) Increased number of re-absorbed embryos (indicated by arrows) at E16.5 from a heterozygous breeding pair. (B) Body weight of *Etv5^sco/sco^* and WT mice at 3 weeks and 3 months. Data are shown as mean ± standard deviation (S.D.), *n* = 6 per genotype, *p<0.05, **p<0.01 (unpaired *t*-test, two-tailed). (C) Renal asymmetry (D) and polydactyly in adult (12 weeks-old) *Etv5^sco/sco^* mice.

To further define the precise time point of pup loss, we collected embryos from heterozygous intercrosses at embryonic day 10.5 through to 18.5 (E10.5–E18.5) and genotyped them to compare the frequency of wild-type (*Etv5^WT/WT^*), heterozygous (*Etv5^WT/sco^*) and homozygous (*Etv5^sco/sco^*) progeny. If no embryonic lethality was occuring, the frequencies of progeny would be predicted to correspond with the Mendalian distribution i.e. approximately 50%, 25% and 25% of progeny would be *Etv5^WT/sco^*, *Etv5^WT/WT^* and *Etv5^sco/sco^*, respectively. At E10.5–13.5, the frequency of all genotypes was not significantly different from the expected Mendalian distribution (Table 1). The frequency of *Etv5^sco/sco^* was however, was significantly reduced at E14.5–18.5 i.e. only 15.3% of all embryos screened were *Etv5^sco/sco^* (Table 1). These results suggest that ∼10% of *Etv5^sco/sco^* embryos were lost during the late gestation period. Consistent with these results, we found an increased incidence of pre-absorption sites at E14.5–E18.5 (Fig. 4A). At present, it is unknown if embryonic loss is the result of an inherent embryonic defect or if it is related to placental insufficiency.

Following birth, there was a further reduction in the frequency of *Etv5^sco/sco^* to 10.7% and 8.3% at 1 and 3 weeks after birth, respectively. These data strongly suggest that ETV5 plays a critical role in the survival of foetal and newborn pups. The reasons for this loss are currently unknown.

### Postnatal Growth Restriction, Renal Asymmetry and Polydactyly in Etv5^sco/sco^ Mice

To further define the role of ETV5 in postnatal development, we monitored body weight of *Etv5^sco/sco^* mice and WT (*Etv5^WT/WT^*) littermates at weaning age (3 weeks) and 3 month-of-age. There was no significant difference in body weights between *Etv5^WT/WT^* and *Etv5^WT/sco^* mice at either age (Fig. 4B). Body weight in *Etv5^sco/sco^* mice was however, significantly reduced compared to *Etv5^WT/WT^* and *Etv5^WT/sco^* littermates by 3 week-of-age (Fig. 4B). By 3 months, *Etv5^sco/sco^* body weight was reduced by ∼50% compared to of *Etv5^WT/WT^* littermates (Fig. 4B). These findings suggest the *Etv5* mutation compromised the development of many organs and ultimately led to postnatal growth restriction.

In addition, approximately 33% (5 out of 15) and 53% (8 out of 15) of *Etv5^sco/sco^* mice (Table 2) that survived to adulthood exhibited renal asymmetry (Fig. 4C) and polydactyly, respectively (Fig. 4D). These results are consistent with previous studies that demonstrated an essential role for ETV5 and its closely related paralog, ETV4, in kidney development [16] and limb patterning [17], [18].

**Table 2:** Incidence of developmental abnormalities in *Etv5^sco/sco^* adult mice.

Genotype	Total number of mice examined	No. of mice with reducedbody weight	No. of mice with renal asymmetry	No. of mice with polydactyly (%)
*Etv5^WT/WT^*	15	0	0	0
*Etv5^WT/sco^*	18	0	1 (6%)	0
*Etv5^sco/sco^*	15	15 (100%)	5 (33%)	8 (53%)

### Allelic Series of Etv5 Mouse Models Give Rise to Phenotypes of Differing Severity

**Figure 5:**
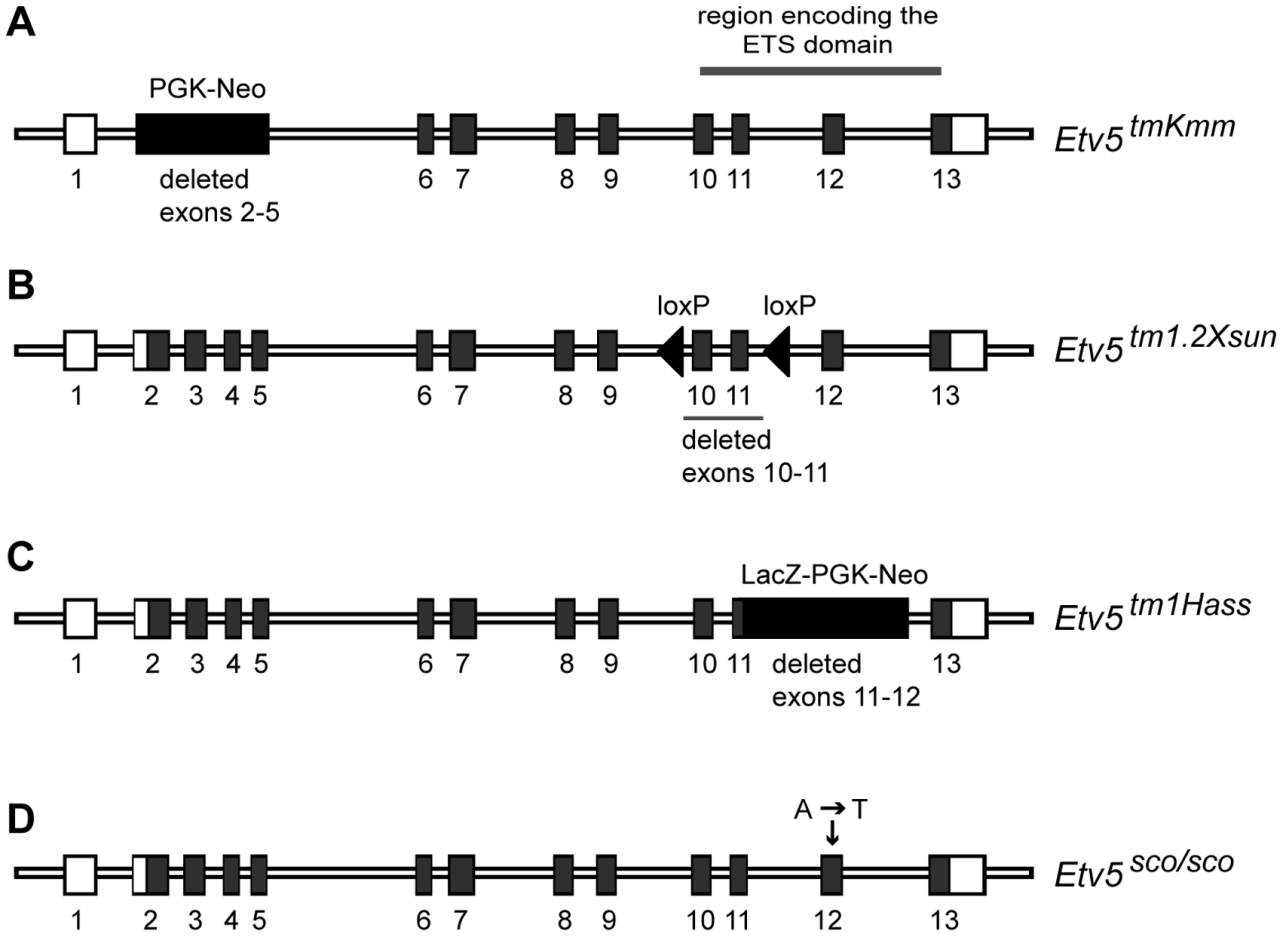
Allelic series of *Etv5* mouse models. (A) The *Etv5^tmKmm^* mouse line carried a targeted deletion of exons 2–5. (B) *Etv5^tm1.2Xsun^* allele mouse line carried a targeted deletion of exons 10–11. (C) The *Etv5^tm1Hass^* mouse line carried a targeted deletion of exons 11–12 which were replaced with a LacZ-PGK-Neo cassette. (D) *Etv5^sco/sco^* mouse line contained an ENU-induced PTC-mutation in exon 12.

Three additional allelic variants of *Etv5* mice have been generated 7,16,17 (Fig. 5). The first mouse line carried a targeted deletion of exons 2–5 of the *Etv5* gene (referred to as *Etv5^tm1Kmm^*) [7] (Fig. 5A, Table 3). This targeted allele resulted in the production of a truncated mRNA containing exons 1 fused to exons 6–13 (Fig. 5A) and the production of a frame-shifted mRNA. Using an antibody against a region encoded by exons 7 and 8, no protein was detected in the *Etv5^tm1Kmm^* homozygous adult testis [7]. The second mouse line (referred to as *Etv5^tm1.2Xsun^*) was generated by targeted insertion of loxP sites flanking the EST domain-coding exons 10 and 11 (Fig. 5B). Excision of floxed exons was achieved using a ubiquitous cre transgenic line [17]. This allele produced highly unstable *Etv5* mRNA i.e. <2% compared to the *Etv5* floxed allele. The third *Etv5* targeted allele (refers to as *Etv5^tm1Hass^*) was generated by replacing the ETS domain-coding exons 11–12 with an IRES-NLS-LacZ-PGK-neo cassette [16] (Fig. 5C). Our SCO mouse line is the fourth in the *Etv5* allelic series and carries a missense mutation in the ETS domain-coding exon 12 (Fig. 5D) that resulted in the production of highly unstable mRNA and a resultant loss of detectable protein (Fig. 3D).

**Table 3:** Phenotypic defects observed in homozygous mice from the *Etv5* allelic series.

Model	*Etv5^tm1Kmm^* [7]	*Etv5^tm1.2Xsun^* [17]	*Etv5^tm1Hass^* [16]	*Etv5^sco/sco^*
Deleted exons	Exons 2–5 (including theinitiation codon)	Exons 10 and 11	Part of exon 11 through to intron 12 was replaced with a LacZ cassette	PTC-mutation in exon 12
Effects on mRNA and protein	Truncated mRNA (exons fused to exons 6–13); frame-shifted but stable mRNA; no protein was detected.	Truncated mRNA (missing exonsencoding the ETS domain);frame-shifted and highlyunstable mRNA; the presenceof the truncated proteinwas not determined.	Truncated mRNA (missing exons encoding the ETS domain); the presence of the truncated protein was not determined.	Truncated mRNA (missing regions encoding the 99 amino acids C-terminal of the ETS domain); highly unstable mRNA (<5% of the wild-type level); no protein was detected.
Embryonic/Perinatal lethality	No defects	Majority of mice diedshortly after birth	Embryonic lethality	Increased embryonic and perinatal lethality
Male fertility defects	Sterile; exhibited Sertoli cell only phenotype at 10 weeks	n/a	n/a	Sterile; exhibited Sertoli cell only phenotype at 8 weeks
Female fertility defects	Sterile, defects in oocyte development, decreased ovulation and mating rates	n/a	n/a	Sterile
Effects on body weight	100% of mice had reduced body weight weights	n/a	n/a	100% of mice that survived to adulthood had reduced body weights
Renal asymmetry	No defects	n/a	n/a	33% of mice that survived to adulthood had renal asymmetry
Polydactyly	No defects	n/a	n/a	53% of mice that survived to adulthood had polydactyly
Genetic background	129S6/SvEvTac	129S1/Sv*129X1/SvJ*C57BL/6*SJL	129S1/Sv	C57BL/6J × CBA

n/a: not analysed due to embryonic lethality.

The four *Etv5* mouse lines exhibited some overlapping phenotypic defects but with varying degrees of severity as summarised in Table 3. We noted that deletions of exons encoding the ETS domain in *Etv5^tm1.2Xsun^*, *Etv5^tm1Hass^* and our *Etv5^sco/sco^* mouse lines resulted in a more severe phenotype i.e. embryonic and perinatal lethality (Table 3), whereby deletion of exons 2–5 in the *Etv5^tm1Kmm^* mouse line did not compromise viability. The mechanism that drives this varying phenotypic severity is currently unknown.

In summary, we have further confirmed the essential role ETV5 plays in fertility. Moreover, our data have defined a physiological role for ETV5 in embryonic and perinatal survival, postnatal growth, limb patterning and kidney development.

## Materials and Methods

### ENU Mutagenesis

All animal experiments were approved by the Australian National University (ANU) and the Monash University Animal Ethics Committees. ENU mutagenesis was performed at the Australian Phenomics Facility (Canberra, Australia) as previously described [19]–[21]. Phenotypic screens for sterile mouse lines were performed as previously described [19], [27].

### Mutation Identification

Linkage analysis was performed to localize the chromosomal region responsible for the phenotype using single nucleotide polymorphisms (SNPs) that differ between the C57BL/6 and CBA strains. This was performed using Affymetrix GeneChip Mouse Mapping 5K SNP Chip (Affymetrix) at the Australian Genome Research Facility (AGRF, Brisbane, Queensland, Australia). The identified linkage interval was further narrowed using the Amplifluor SNP detection system (Chemicon) on additional affected and unaffected mice. Candidate gene sequencing was performed at the AGRF.

### Mice Genotyping

Genotyping was performed using the Amplifluor SNP detection system as previously described [20], [21]. Primers used included a sense primer, SCO-Fw: 5′-CTGTCTTGTCCTTCCAGGTT-3′ and a wild-type specific antisense primer, SCO-WT: 5′-GAAGGTCGGAGTCAACGGATTGTTCATGGCTGGCCGATTCTT-3′, and a mutant specific antisense primer, SCO-mutant: 5′-GAAGGTGACCAAGTTCATGCTGTTCATGGCTGGCCGATTCTA-3′. PCR amplification was carried out as follows: 1 cycle at 95°C, 4 min, 35 cycles of (95°C for 10 sec, 60°C for 20 sec, 72°C for 40 sec), 1 cycle at 72°C, 3 min. After amplification, the plates are read in a BMG Fluostar optima fluorescent microplate reader (BMG Labtech).

### Quantitative PCR Analysis

Total RNA samples were isolated using Trizol reagent (Invitrogen) according to the manufacturer’s instruction. Approximately 2 µg of total RNAs were converted to cDNAs using SuperScriptIII reverse transcriptase (Invitrogen). *Etv5*, *Ccl9* and *Cxcr4* mRNA expression was determined by quantitative PCR using TaqMan Gene Expression Assays (Applied Biosystems), assay IDs Mm00465816_m1, Mm00441260 and Mm019964749, respectively. Reference genes were *Hprt* (assay ID Mm00446968_m1), *Ppia* (assay ID Mm02342429) and *Idh2* (assay ID Mm02342429_g1).

### ETV5 Immunoblotting

A rabbit polyclonal antibody raised against amino acids 121–220 of human ETV5 was used for the detection of the ETV5 protein (sc-22807, Santa Cruz Biotechnology). Immunoblotting blot analysis was performed as previously described [28] using 2 µg/ml of the ETV5 antibody. HPRT was used as a loading control (Abcam, ab10479).
